# Analysis of risk factors associated with Lymph Node Metastasis and Recurrence Post Thyroid Carcinoma Surgery

**DOI:** 10.12669/pjms.41.6.10705

**Published:** 2025-06

**Authors:** Shengchao Wang, Zhongxu Bai, Huijuan Yan, Chunmei Yan, Junhui Wang

**Affiliations:** 1 Shengchao Wang Department of Thyroid Surgery, Yellow River Sanmenxia Hospital, Sanmenxia 472000. Henan, Chian; 2 Zhongxu Bai Department of Orthopaedics, Zhengzhou Second Hospital, Zhengzhou 450052, Henan, China; 3 Huijuan Yan Department of Gynecology, Yellow River Sanmenxia Hospital, Sanmenxia 472000. Henan, Chian; 4 Chunmei Yan Department of Hematology and Oncology, The 988th Hospital of the Joint Logistics Support Force, People’s Liberation Army of China, Zhengzhou 450000, Henan, China; 5 Junhui Wang Department of Thyroid Surgery, Yellow River Sanmenxia Hospital, Sanmenxia 472000. Henan, Chian

**Keywords:** Lymph node metastasis, Recurrence, Risk factors, Survival analysis, Thyroid carcinoma

## Abstract

**Objective:**

To investigate the risk factors associated with postoperative lymph node metastasis and recurrence in patients with thyroid carcinoma.

**Methods:**

Retrospective analysis of clinical data of one hundred patients with thyroid carcinoma who underwent first surgery at the Yellow River Sanmenxia Hospital from June 2019 to July 2023. Record postoperative lymph node metastasis, recurrence, and disease-free survival time. Kaplan Meier survival analysis was used to evaluate the recurrence free survival rate after surgery. Compared the differences between groups with and without lymph node metastasis. Analysis of risk factors for postoperative lymph node metastasis and recurrence in patients with thyroid carcinoma.

**Results:**

In study, 27 out of 100 patients experienced lymph node metastasis and recurrence, with an overall recurrence rate of 27.00%. There were nine cases of intrathyroid recurrence, 15 cases of cervical lymph node metastasis, two cases of intrathyroid metastasis with cervical lymph node metastasis, and one case of lung metastasis. Univariate analysis showed that gender, age, history of hyperthyroidism, tumor classification and staging, tumor diameter, number of cancer foci, unilateral or bilateral lesions, ^131^I treatment, and surgical methods were influencing factors for postoperative lymph node metastasis and recurrence (*P*<0.05). Logistic regression analysis identified gender, age, history of hyperthyroidism, tumor classification and stage, unilateral or bilateral disease involvement as independent risk factors for postoperative lymph node metastasis and recurrence in thyroid carcinoma (*P*<0.05).

**Conclusion:**

Gender, age, history of hyperthyroidism, tumor classification, tumor staging, and unilateral or bilateral lesions are independent risk factors for postoperative lymph node metastasis and recurrence in thyroid carcinoma.

## INTRODUCTION

Thyroid carcinoma, a prevalent endocrine and malignant thyroid tumor in clinical settings, has seen an increasing incidence rate over the years^1^, particularly among women. In developed regions such as China, Japan, and South Korea, the incidence of thyroid carcinoma has multiplied in recent decades.^2^ In 2012, China accounted for 15.6% of the world’s new thyroid carcinoma cases and 13.8% of deaths.^3^ The incidence rate of thyroid carcinoma in China in 2016 was 10.58 per 100,000, with a male-to-female ratio of 1:3.2, placing thyroid carcinoma as the third most common cancer among women.^4^ Data indicated that the 20-years survival rate for thyroid carcinoma patients in the United States reached 97%, the five-years survival rate in Europe was 86.5%, while the five-years survival rate in China was 84.3%, showing a disparity with developed countries.^5^

The etiology of thyroid carcinoma remains unclear. Although it can occur at any age, it predominantly affects young and middle-aged adults. Once diagnosed, aggressive surgical resection is the primary treatment method, yet the risk of metastasis and recurrence post-surgery persists. Patients and their families are deeply concerned about surgical outcomes, postoperative prognosis, and survival. Unfavorable surgical results or postoperative metastasis/recurrence can cause significant distress to patients and their families. Studies have verified that gender, age, and thyroid nodules are risk factors for thyroid carcinoma^6^, yet the factors influencing lymph node metastasis and recurrence post-surgery remain controversial. Some research indicates that male gender, age, tumor size, and lymph node metastasis are independent risk factors for postoperative recurrence of thyroid carcinoma^7^, while other studies suggest gender is not a risk factor.^8^ Current knowledge on the factors affecting cervical lymph node metastasis and postoperative recurrence of thyroid carcinoma primarily comes from international studies, with limited domestic research. Hence, this study’s primary goal is to clarify the factors influencing cervical lymph node metastasis and postoperative recurrence of thyroid carcinoma in the Chinese population, which holds significant importance for guiding the treatment of thyroid carcinoma in China.

## METHODS

A retrospective analysis was conducted on the clinical data of one hundred patients diagnosed with thyroid carcinoma and undergoing initial surgery at Yellow River Sanmenxia Hospital from June 2018 to July 2023. Lymph node metastasis was confirmed by routine pathology or clinical diagnosis of distant bone or lung metastasis. Recurrence was confirmed by biopsy or pathological examination of the reappearing tumor site. Patient information was collected from the hospital’s medical record system, including gender, age, history of hyperthyroidism, tumor classification, tumor staging, tumor diameter, number of cancer foci, unilateral or bilateral lesions, surgical methods, and ^131^I treatment. Patients were followed up for five-years through outpatient reexaminations, WeChat, or telephone contact.

A total of one hundred thyroid cancer patients were screened from June 2018 to June 2023 according to inclusion and exclusion criteria, including 28 males and 72 females, aged 32-78 years. Among them, there were 88 cases of papillary carcinoma, six cases of follicular carcinoma, four cases of medullary carcinoma, and two cases of undifferentiated carcinoma. During the follow-up period, 27 cases experienced recurrence and metastasis, while 73 cases did not. Specifically, there were nine cases of intrathyroidal recurrence, 15 cases of cervical lymph node metastasis, two cases of intrathyroidal recurrence with cervical lymph node metastasis, and one case of distant metastasis (lung metastasis). From the date of surgery to the end of follow-up on July 30, 2023, the follow-up period ranged from one to 60 months, during which 27 patients(27.00%) experienced recurrence.

### Ethics Approval:

The study was approved by the Institutional Ethics Committee of Yellow River Sanmenxia Hospital (No.: 2021-0901; date: September 01,2021), and written informed consent was obtained from all participants.

### Inclusion criteria:

- Patients who underwent initial thyroidectomy and were diagnosed with thyroid carcinoma at our hospital from June 2018 to July 2023.
- Complete clinical data available to determine whether the patient had metastasis and recurrence.
- Effective control of metastasis after treatment if it occurred.

### Exclusion criteria:

- Patients with other malignant tumors.
- Patients with severe heart, lung, or kidney dysfunction.

### Observation and evaluation indicators:

During the follow-up period, lymph node metastasis and recurrence were recorded. Based on relevant literature, this study selected factors such as gender, age, body mass index, history of hyperthyroidism, tumor classification, tumor staging, tumor diameter, number of cancer foci, unilateral or bilateral lesions, surgical methods, and ^131^I treatment to analyze the influencing factors of cervical lymph node metastasis and postoperative recurrence of thyroid carcinoma.

### Statistical analysis:

All data were statistically analyzed using the SPSS 21.0 software (SPSS Inc., Chicago, IL, USA). Measurement data were expressed as mean ± standard deviation (X¯±S), and t-tests were used for comparisons between groups. Enumeration data were expressed as numbers and percentages[n (%)], and comparisons between groups were made using χ^2^ test or Fisher’s exact probability method. Logistic regression analysis was used to identify influencing factors. *P*-value <0.05 was considered a statistically significant difference.

## RESULTS

**Fig.1:**
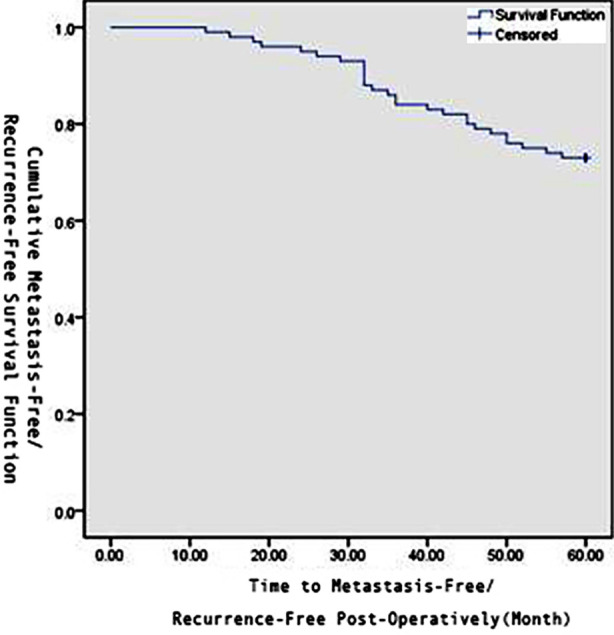
Cumulative Recurrence-Free Survival Function Post Thyroid Carcinoma Surgery.

The Kaplan-Meier method was used to derive the one year, three years, and five years recurrence-free survival curves for thyroid cancer patients postoperatively, as shown in Fig.1. Univariate analysis was conducted on various factors that might influence lymph node metastasis and postoperative recurrence in thyroid cancer patients. The results showed that gender, age, history of hyperthyroidism, tumor type, tumor stage, tumor diameter, number of cancer foci, unilateral or bilateral lesions, ^131^I treatment, and surgical method were all influencing factors for lymph node metastasis and postoperative recurrence in thyroid cancer patients(*P*<0.05), Table-I. Based on the results of univariate analysis, gender, age, history of hyperthyroidism, tumor type, tumor stage, tumor diameter, number of cancer foci, unilateral or bilateral lesions, ^131^I treatment, and surgical method were identified as risk factors for lymph node metastasis and postoperative recurrence in thyroid carcinoma patients. To exclude the mutual influence of these risk factors and further identify independent risk factors, the above 11 risk factors were included in the Logistic regression model for multivariate analysis. The results showed that gender, age, history of hyperthyroidism, tumor type, tumor stage, and unilateral or bilateral lesions were independent risk factors for lymph node metastasis and postoperative recurrence in thyroid carcinoma patients (*P*<0.05), Table-II.

**Table-I:** Univariate Analysis of Lymph Node Metastasis and Postoperative Recurrence in Thyroid Cancer [n(%)].

Variable	Recurrence/ Metastasis Group	Non-Recurrence/ Metastasis Group	χ^2^	P
Gender			5.233	0.022
Male	3 (11.11)	25 (34.25)		
Female	24 (88.89)	48 (65.75)		
Age (years)			4.619	0.032
≥45	18 (66.67)	31 (42.47)		
<45	9 (33.33)	42 (57.53)		
Body Mass Index (kg/m^2^)			0.457	0.499
≥24	15 (55.56)	35 (47.95)		
<24	12 (44.44)	38 (52.05)		
History of hyperthyroidism			4.822	0.028
Yes	17 (62.96)	28 (38.36)		
No	10 (37.04)	45 (61.64)		
Tumor Type			8.280	0.041
Medullary carcinoma	9 (33.33)	10 (13.70)		
Papillary carcinoma	14 (51.86)	55 (75.34)		
Follicular carcinoma	3 (11.11)	8 (10.96)		
Undifferentiated carcinoma	1 (3.70)	0 (0.00)		
Tumor stage			12.412	0.004
Stage I	4 (14.81)	29 (39.73)		
Stage II	4 (14.81)	22 (30.14)		
Stage III	12 (44.45)	15 (20.54)		
Stage IV	7 (25.93)	7 (9.59)		
Tumor diameter (cm)			3.989	0.046
≥2	16 (59.26)	27 (36.99)		
<2	11 (40.74)	46 (63.01)		
Number of cancer foci			8.126	0.004
Single	10 (37.04)	50 (68.49)		
Multiple	17 (62.96)	23 (31.51)		
Unilateral or bilateral lesions			4.261	0.039
Unilateral	12 (44.44)	49 (67.12)		
Bilateral	15 (55.56)	24 (32.88)		
^131^I treatment			5.463	0.019
Yes	15 (55.56)	22 (30.14)		
No	12 (44.44)	51 (69.86)		
Surgical method			5.716	0.017
Total thyroidectomy	11 (40.74)	49 (67.12)		
Subtotal thyroidectomy	16 (59.26)	24 (32.88)		

**Table-II:** Multivariate logistic regression analysis of factors influencing lymph node metastasis and postoperative recurrence of thyroid carcinoma.

Factor	Regression Coefficient	Standard Error	Wald c^2^	P	OR	95%CI
Gender	-8.551	2.929	8.523	0.004	0.000	6.209E-7-0.060
Age	3.967	1.569	6.397	0.011	52.851	2.443-1143.535
History of hyperthyroidism	3.109	1.325	5.507	0.019	22.397	1.669-300.524
Tumor type	-12.264	2.866	18.313	0.000	4.719E-6	1.716E-8-0.001
Tumor stage	-4.337	1.876	5.344	0.021	0.013	0.000-0.517
Unilateral or bilateral lesions	-2.729	1.358	4.036	0.045	0.0065	10.005-0.935

## DISCUSSION

In this study, the postoperative cervical lymph node metastasis and recurrence in female patients was significantly higher than in male patients, making it an independent risk factor for postoperative cervical lymph node metastasis and recurrence(*P*<0.05). Based on the literature, we believe that this phenomenon is related to the presence of estrogen receptor genes in thyroid carcinoma, which exhibit polymorphism. The expression level of estrogen receptor genes in females is higher than in males, which may explain the gender differences in the incidence of thyroid carcinoma. This also suggests that hormonal and reproductive factors are risk factors for postoperative cervical lymph node metastasis and recurrence in females. However, some studies have shown that the recurrence rate in males is higher than in females^9^,^10^, while other studies have shown that gender differences are not related to cervical lymph node metastasis and recurrence after thyroidectomy.^11^

The results of this study indicated that the postoperative cervical lymph node metastasis and recurrence rate in patients aged ≥4 five-years is significantly higher than in those aged <4 five-years (*P*<0.05), making it an independent risk factor for postoperative cervical lymph node metastasis and recurrence (*P*<0.05). Currently, there is still controversy regarding the age cutoff for TNM staging of thyroid carcinoma. Some studies suggest that age is an important factor influencing the prognosis of thyroid carcinoma within the age range of 25-5 five years, regardless of the age used as the grouping cutoff.^12^ Other studies have shown^13^ that the age at first diagnosis is a factor influencing cervical lymph node metastasis and recurrence after thyroidectomy, with ≥4 five-years being a risk factor for postoperative cervical lymph node metastasis and recurrence. However, some studies have shown that age is not related to cervical lymph node metastasis and recurrence after thyroidectomy.^14^

Studies have shown that some patients with hyperthyroidism may develop thyroid carcinoma.^15^ The relationship between hyperthyroidism and thyroid carcinoma remains controversial, and its pathogenesis is still unclear. Current research suggests that hyperthyroidism may influence the occurrence of thyroid carcinoma. This is because long-term excessive use of antithyroid drugs in hyperthyroid patients can lead to elevated thyroid-stimulating hormone (TSH) levels, which is a promoting factor for the development of thyroid carcinoma.^16^ Our study results indicate that among 27 patients with postoperative metastasis/recurrence of thyroid carcinoma, 17 had a history of hyperthyroidism. Both univariate and multivariate analyses showed that hyperthyroidism was statistically significant (*P*<0.05) as a risk factor for postoperative metastasis/recurrence of thyroid carcinoma.

Therefore, for patients with thyroid carcinoma combined with hyperthyroidism, reasonable postoperative treatment, regular check-ups, and follow-ups are necessary to prevent postoperative metastasis/recurrence of thyroid carcinoma. Staging and classification of thyroid carcinoma play a significant role in predicting postoperative prognosis, metastasis, and recurrence in thyroid carcinoma patients. Previous studies have confirmed^17^ that the later the TNM stage, the higher the rate of postoperative metastasis/recurrence. Our study results show that among the 27 patients with metastasis/recurrence, 12 were in Stage-III and seven in Stage-IV. Univariate and multivariate analyses indicated that the TNM stage of thyroid tumors was statistically significant (*P*<0.05) as an independent risk factor for postoperative metastasis/recurrence, consistent with previous research findings.^18^ The pathological type of thyroid carcinoma not only guides treatment but also predicts patient prognosis. Studies have shown that different pathological types of thyroid carcinoma have varying impacts on prognosis.^19^ High-grade thyroid carcinomas (such as medullary carcinoma and anaplastic carcinoma) are associated with a significantly increased risk of postoperative metastasis/recurrence due to their higher invasiveness and metastatic potential. Our study’s univariate and multivariate analyses revealed that the pathological type of thyroid carcinoma was statistically significant (*P*<0.05) as an independent risk factor for postoperative metastasis/recurrence, consistent with previous studies.^20^

Additionally, whether the tumor is unilateral or bilateral is also an important factor in predicting postoperative metastasis/recurrence of thyroid carcinoma. Studies have found^21^ that the presence of bilateral primary tumors is associated with an increased risk of postoperative metastasis/recurrence. This may be due to the greater extent of tumor spread and lymph node involvement in bilateral lesions compared to unilateral lesions, as well as differences in treatment approaches, which directly affect treatment outcomes and prognosis. Our study’s univariate and multivariate analyses showed that unilateral or bilateral lesions were statistically significant (*P*<0.05) as independent risk factors for postoperative metastasis/recurrence of thyroid carcinoma.

### Limitations:

It includes a small number of observed cases and limited follow-up factors. Future studies should expand the sample size and increase follow-up factors to further analyze the factors influencing postoperative metastasis/recurrence of thyroid carcinoma.

## CONCLUSIONS

Postoperative metastasis/recurrence of thyroid carcinoma is a critical issue that needs attention in the treatment of thyroid carcinoma. Factors such as gender, age, history of hyperthyroidism, tumor classification, tumor staging, and unilateral or bilateral lesions are major risk factors associated with postoperative metastasis/recurrence of thyroid carcinoma. In-depth research and understanding of these factors are crucial for improving the treatment outcomes and prognosis of thyroid carcinoma.

### Authors’ Contributions:

**SW:** Carried out the studies, participated in collecting data, and drafted the manuscript, and is responsible and accountable for the accuracy or integrity of the work.

**ZB, HY, CY** and **JW:** Study design**,** Performed the statistical analysis and critical review.

All authors have read and approved the final manuscript.

## References

[1] Wang LL, Li HQ, Chang QG, Li S, Yin DT (2020). Clinical pathology and incidence trend of thyroid cancer based on 21980 cases. Zhonghua Yi Xue Za Zhi.

[2] Siano M, Alfieri S, Granata R, Calareso G, Orlandi E, Bergamini C, Locati LD (2019). The dilemma of metastatic medullary thyroid carcinoma:when to start systemic treatment. Tumori.

[3] Amin SN, Shinn JR, Naguib MM, Netterville JL, Rohde SL (2020). Risk Factors and Outcomes of Postoperative Recurrent Well-Differentiated Thyroid Cancer:A Single Institution's 1five-years'Experience. Otolaryngol Head Neck Surg.

[4] Shah AA, Jain PP, Dubey AS, Panjwani GN, Shah HA (2018). A study of clinicopathological characteristics of thyroid carcinoma at a Tertiary Care Center. J Cancer Res Ther.

[5] Liu J, Liu X, Guo Z, Lv X, Mao W, Xu D (2021). Ultrasound-guided fine needle aspiration cytology of Para-aortic lymph node metastasis in uterine cervical cancer:diagnostic accuracy and impact on clinical decision making. BMC Cancer.

[6] Lim ST, Jeon YW, Gwak H, Suh YJ (2020). Incidence, Risk Factors, and Clinical Implications of Delayed Hypoparathyroidism on Postoperative Day two Following Total Thyroidectomy for Papillary Thyroid Carcinoma. Endocr Pract.

[7] De Jong MC, Gaze MN, Szychot E, Rozalén García V, Brain C, Dattani M (2021). Treating papillary and follicular thyroid cancer in children and young people:Single UK-center experience between, 2003 and 2018. J Pediatr Surg.

[8] Luo J, Li H, Deziel NC, Huang H, Zhao N, Ma S (2020). Genetic susceptibility may modify the association between cell phone use and thyroid cancer:A population-based case-control study in Connecticut. Environ Res.

[9] Siraj AK, Parvathareddy SK, Annaiyappanaidu P, Siraj N, Al-Sobhi SS, Al-Dayel F (2022). Male Sex Is an Independent Predictor of Recurrence-Free Survival in Middle Eastern Papillary Thyroid Carcinoma. Front Endocrinol (Lausanne).

[10] Shen W, Pan XJ, Li QH (2022). Utility and significance of clinical risk factor scoring model in predicting central compartment lymph node metastasis (CLNM) in patients with papillary thyroid cancer (PTC). Pak J Med Sci.

[11] Bae SY, Jung SP, Choe JH, Kim JS, Kim JH (2019). Prediction of lateral neck lymph node metastasis according to preoperative calcitonin level and tumor size for medullary thyroid carcinoma. Kaohsiung J Med Sci.

[12] Marsden M, Weaver SS, Marcu L, Campbell MJ (2021). Intraoperative Mapping of Parathyroid Glands Using Fluorescence Lifetime Imaging. J Surg Res.

[13] Su A, Zhao W, Wu W, Wei T, Ruan M, Li Z (2020). The association of preoperative thyroid-stimulating hormone level and the risk of differentiated thyroid cancer in patients with thyroid nodules:A systematic review and meta-analysis. Am J Surg.

[14] Zhao J, Wang H, Zhang Z, Zhou X, Yao J, Zhang R (2019). Vitamin D deficiency as a risk factor for thyroid cancer:A meta-analysis of case-control studies. Nutrition.

[15] Xia M, Zang J, Cheng H, Song J, Wang Z, Zhu H (2021). Case-control study on relationship between diet quality and papillary thyroid carcinoma. J Environ Med.

[16] Miyauchi A, Kudo T, Kihara M, Oda H, Ito Y, Miya A (2019). Spontaneous Deceleration and Acceleration of Growth Rate in Medullary Thyroid Carcinomas Suggested by Changes in Calcitonin Doubling Times Over Long-Term Surveillance. World J Surg.

[17] Means C, Clayburgh DR, Maloney L, Sauer D, Taylor MH, Shindo ML (2019). Tumor immune microenvironment characteristics of papillary thyroid carcinoma are associated with histopathological aggressiveness and BRAF mutation status. Head Neck.

[18] Choi JB, Lee SG, Kim MJ, Kim TH, Ban EJ, Lee CR (2019). Oncologic outcomes in patients with 1-cm to 4-cm differentiated thyroid carcinoma according to extent of thyroidectomy. Head Neck.

[19] Han MA, Kim JH, Song HS (2019). Persistent organic pollutants, pesticides, and the risk of thyroid cancer:systematic review and meta-analysis. Eur J Cancer Prev.

[20] Wu T, Liu M, Bai P, Wang Z, Zang J, Guo C (2021). Case-control study on association of female reproductive factors with risk of papillary thyroid cancer. J Environ Occup Med.

